# Prevalence of A2 and A2B Subgroups among Blood Groups A and AB in healthy donors

**DOI:** 10.12669/pjms.40.1.7916

**Published:** 2024

**Authors:** Ayesha Khanum, Saima Farhan, Nazish Saqlain, Sundas Arshad

**Affiliations:** 1 Ayesha Khanum, FCPS. Consultant Hematologist, University of Child Health Sciences, The Children’s Hospital, Lahore, Pakistan; 2 Saima Farhan FCPS. Associate Professor of Paeds. Haematology, University of Child Health Sciences, The Children’s Hospital, Lahore, Pakistan; 3 Nazish Saqlain, FCPS. Associate Professor of Pathology, University of Child Health Sciences, The Children’s Hospital, Lahore, Pakistan; 4 Sundas Arshad, FCPS. Consultant Hematologist, Social Security Teaching Hospital, Multan Road, Lahore, Pakistan

**Keywords:** A2, A2B, Blood group, Anti-sera, Blood donors

## Abstract

**Objective:**

To determine the frequency of A2 and A2B subgroups among blood groups A and AB in healthy donors.

**Methods:**

It was a Cross-Sectional study, conducted at the Department of Hematology & Transfusion Medicine, UCHS, The Children’s Hospital Lahore and Sundas foundation Lahore from June 2022 to December 2022 including 13,120 healthy blood donors of both genders, after taking informed consent. Venous blood samples of donors were collected in EDTA vials (3ml) and serum gel vial for routine blood grouping which was done by standard tube method. Further testing of donors positive for an antigen (blood Group-A and AB) was performed using anti-A1 lectin by standard tube method as per manufacturer’s instruction. The data was analyzed using SPSS version 23.

**Results:**

Among 13120 blood donors, 12857 (97.9%) were male and 263 (2.0%) were female with mean age of 36.7 years ± 15.04 years. Majority of them (91.7%) were of Punjabi ethnicity. Donors having blood group phenotype A and AB were 3890 (29.6%). Among blood Group-A donors, A1 was found in 97.8% and A2 in 2.2% donors. While among Blood Group-AB, 96.7% donors belonged to A1B blood group and 3.2% belonged to A2B blood group.

**Conclusions:**

Blood group A2 and A2B do exist in blood donors of Punjabi ethnicity. The knowledge of presence of these blood groups’ phenotypes in our population can provide a better base for transfusion staff to do troubleshooting in compatibility testing and to avoid any rare but hazardous transfusion outcome.

## INTRODUCTION

ABO blood group was introduced by Karl Landsteiner in 1901. This blood group is based on antigens present on RBCs.^1^ With advances of knowledge in science these ABO groups are further divided into subgroups. The blood groups are important in reference to blood transfusions and organ and bone marrow transplants. Major ABO blood groups are A, B, AB and O. Different subcategories of A and B blood groups are identified. A1 and A2 are relatively common subgroups of Blood Group-A. Other less common subtypes of A include A3, Ax, A^end^, A^y^ and A^el^. Subgroups of ‘B’ with decreased expression of the B antigen are of very rare occurrence in the general population. Serologically, the variants of ‘B’ can be classified into B3, B^x^, B^m^ and B^el^. Serological confirmation of such subgroups requires special immuno-hematological procedures.^2^ A1 and A2 are different in structure and function. A1 red cells express approximately five times more A antigen than A2 red cells, but both types of red cell react with anti-A.^3^

These subgroups can be distinguished from each other by reacting with Anti-A1 Lectin which occurs as a cold agglutinin and reacts with A1 cells. Anti-A1 lectin obtained from Dolichos Biflorus seeds is routinely used in blood banks to differentiate between A1 and A2 red blood cells.^4^ Persons having Blood Group-A2 and A2B may have anti-A1 antibody. Usually, anti-A1 exists as cold antibody which is naturally occurring, with a thermal amplitude of less than 25°C. However, cases of anti-A1 reacting at 37° C have also been reported in the literature.^2^ The A1 and A2 subgroups are different from each other both qualitatively and quantitatively, with A1 red cells having 8.1 - 11.7×10^5^ antigenic sites as compared to 2.4–2.9×10^5^ antigenic sites on A2 red cells. In routine testing, both A1 and A2 are strongly agglutinated by anti-A antiserum but problem arises when A2 blood group individuals develop anti-A1 antibody which can cause discrepancies in blood grouping.^5^

When anti-A1 is active at body temperature, though rare, extensive destruction of A1 cells in vivo can occur and has been documented to cause severe hemolytic transfusion reaction. It is also important in cases of organ transplant.^6^ There are case reports which show that patient having anti A1 antibody had severe hemolytic transfusion reaction and resulted in death of patient.^7^ In this regard, there is no published study focusing central and southern Punjab province population of Pakistan. However, studies were done in Northern Pakistan recently with the same objective.^8^ So, the present study will help to establish the prevalence of A2 subgroup in our region of the province Punjab, which will help to establish the need of A2 testing as a routine blood banking procedure.

## METHODS

This multi-centered Cross-Sectional study was conducted at the Department of Hematology & Transfusion Medicine, The Children’s Hospital and UCHS and Sundas Foundation, Lahore, Pakistan from June 2022 to December 2022. After taking informed consent, 13120 blood donors of both genders were enrolled in the study consecutively. Detailed medical history was taken on a pre-designed hospital donor assessment form, based on WHO (World Health Organization) blood donor selection criteria. Venous blood samples of participants were collected in EDTA vial (3ml) and serum gel vial for routine blood grouping which was done by standard tube method. Forward blood grouping was done by using Anti-sera A, B, AB and D (Lorne) while reverse blood grouping was performed with known A1, B and O cells.

Further testing of samples positive for an antigen (Blood Group-A and AB) was performed using anti-A1 lectin (Lorne) by standard tube method as per manufacturer’s instructions. The Blood Group-A samples giving positive reaction with Anti A1 lectin were labelled as A1 subgroup and Blood Group-AB samples showing positive reaction were considered A1B subgroup. The samples which gave negative result were labelled as A2 and A2B subgroups. The data was analyzed using SPSS version 23. The quantitative variables like age are presented as mean ± 2SD. The qualitative variables are presented as frequencies.

### Ethical Approval

Approval was obtained from the Institutional Review Boards of both centers, (Ref.: 583/CH-UCHS; dated 16/05/2022).

## RESULTS

Out of 13120 blood donors, 12857 (97.9%) were male and 263 (2.0%) were female with male to female ratio of 49:1. The mean age ± 2SD was 36.7 years ± 15.04 years. Majority of them (91%) were of Punjabi origin with 66.2% donors were residents of Lahore (Table-I).

**Table-I:** Demographic details of the donors.

Characteristics	Number *(N)*	Percentage (%)
***Age***		
17-25 years	4,972	37.8
26-50 years	8,148	62.1
***Gender***		
Male	12857	97.9
Female	263	2.0
***Ethnicity***		
Punjabi	11,939	91
Seraiki	854	6.5
Pakhtun	170	1.3
Sindhi	157	1.2
Total	13,120	100

Donors having blood group phenotype A and AB were 3890 (29.6%). Among Blood Group-A donors, A1 was found in 97.8% and A2 in 2.2% donors. While among Blood Group-AB, 96.7% donors belonged to A1B blood group and 3.2% belonged to A2B blood group (Table-II).

**Table-II:** Distribution of A and AB subgroups among blood group A and AB.

ABO phenotype	Sub-Group	Number (N)	Percentage (%)
A	A1	2758	97.8
	A2	62	2.19
	Total	2820	100
AB	A1B	1035	96.7
	A2B	35	3.2
	Total	1070	100

## DISCUSSION

Prevalence of blood groups varies in different parts of the world. Even in one country the frequencies may vary according to geography and ethnicity. Similar is the case in our country with diverse ethnic population.^9^ There are weak subgroups of the Blood Group-A and B, some of which are of clinical importance as they can cause transfusion related adverse events. Moreover, they can be troublesome in routine serological blood bank procedures.^10^ The present study was conducted to see the prevalence of sub groups A1, A2, A1B and A2B among healthy donors. Very few studies are conducted in Pakistan regarding this topic so the local established data is very limited for comparison.

Our study showed the high frequency of A1 existence among Blood Group-A individuals as compared to A2. Similar results are reported by a previous study in respect to high frequency of A1(93%) than A2 (7%).^3^ A study was conducted in North Karnataka, India, according to which A1 was 98.9% and A2 was 1.1% in their respective population. The results are concordant with our study. Similarly, we found that the frequency of Blood Group-A1B and A2B among Blood Group-AB is 96.7% and 3.2% respectively in our study which is similar to other studies done, like one in Odissa India.^11^

The results of our study are different from the study done in North of Pakistan where the prevalence of A2 and A2B is reported higher than our results. This difference may be due to ethnicity and higher ratio of cousin marriages in those areas.^8^ Previous studies done in India showed almost same results with 95.9% of A1 antigen (subgroup A1) and 4.1% had no detectable A1 antigen (subgroup A2) and among AB group individuals 80.8% had A1 antigen (subgroup A1B) and 67(19.2%) had no detectable A1 antigen (subgroup A2B).^12^,^13^ Study done in South India also showed similar results.^14^ Our findings are comparable to the study done by Saboor M et al in Saudia Arabia population showing 2.2% and 0.9% frequency of A2 and A2B blood groups.^2^ However, Elnour MA et al have reported higher frequency of A2 and A2B blood groups in Sudani population.^3^

Another study was done in Rawalpindi Pakistan in which molecular testing of these blood groups was done and according to genotype of blood groups A1 and A2 is 81.7% and 12.9% respectively.^15^ Severe transfusion reactions can occur due to these minor incompatibilities. Implementation of testing of A1 and A2 sub-groups in routine ABO typing is vital for limiting the preventable transfusion related adverse events ensuring recipients’ safety.^16^ This is the first study done in Lahore describing frequencies of A subgroups including such number of participants.

### Limitations

Some of the limitations of the study were time bound sample collection and selection bias in terms of male gender and Punjabi ethnicity representation. For establishment of varying frequency of A2 blood group among different ethnicities, more varied group of study participants would have been beneficial. Females although representing half of our population but their participation in blood donation process has always been scarce in our country.^17^,^18^ Moreover, Anti-A1 antibody testing was not performed. It can be considered as a pilot study and be further extended by performing Anti-A1 antibody testing in people of blood group of A2. Molecular testing for confirmation of these sub-groups can be done but it is not cost effective in under resourced setup.

## CONCLUSION

Blood Group-A2 and A2B exist in blood donors of Punjabi ethnicity of our country in low frequency. However, this acquaintance of the fact can provide a better understanding for troubleshooting while performing compatibility testing and to avoid any hazardous transfusion reactions. Testing of all A and AB blood group samples by A1 lectin can be suggested as a routine blood banks procedure or at least in cases where blood group discrepancy or incompatibility issues arise.

### Authors Contribution:

**AK:** Designed & Contributed in manuscript writing and she will be responsible for the accuracy and maintaining integrity of data. **SF:** Collection & analysis of data. **NS:** Writing and Review of manuscript and references. **SA:** Data Collection, Manuscript writing.
